# A decision-making method for reservoir operation schemes based on deep learning and whale optimization algorithm

**DOI:** 10.3389/fpls.2023.1102855

**Published:** 2023-03-24

**Authors:** Qiang Hu, He-xuan Hu, Zhen-zhou Lin, Zhi-hao Chen, Ye Zhang

**Affiliations:** ^1^ Key Laboratory of Water Big Data Technology of Ministry of Water Resources, Hohai University, Nanjing, China; ^2^ College of Computer and Information, Hohai University, Nanjing, China; ^3^ Office of Teaching Affairs, Nanjing University of Finance and Economics, Nanjing, China

**Keywords:** reservoir operation, decision-making method, convolutional neural network, data augmentation, generative adversarial network, whale optimization algorithm

## Abstract

Reservoir operation is an important part of basin water resources management. The rational use of reservoir operation scheme can not only enhance the capacity of flood control and disaster reduction in the basin, but also improve the efficiency of water use and give full play to the comprehensive role the reservoir. The conventional decision-making method of reservoir operation scheme is computationally large, subjectivity and difficult to capture the nonlinear relationship. To solve these problems, this paper proposes a reservoir operation scheme decision-making model IWGAN-IWOA-CNN based on artificial intelligence and deep learning technology. In view of the lack of data in the original reservoir operation scheme and the limited improvement of data characteristics by the traditional data augmentation algorithm, an improved generative adversarial network algorithm (IWGAN) is proposed. IWGAN uses the loss function which integrates Wasserstein distance, gradient penalty and difference item, and dynamically adds random noise in the process of model training. The whale optimization algorithm is improved by introducing Logistic chaotic mapping to initialize population, non-linear convergence factor and adaptive weights, and Levy flight perturbation strategy. The improved whale optimization algorithm (IWOA) is used to optimize hyperparameters of convolutional neural networks (CNN), so as to obtain the best parameters for model prediction. The experimental results show that the data generated by IWGAN has certain representation ability and high quality; IWOA has faster convergence speed, higher convergence accuracy and better stability; IWGAN-IWOA-CNN model has higher prediction accuracy and reliability of scheme selection.

## 1 Introduction

In recent years, people’s demand for water resources is increasing day by day, and water resources are also facing a series of serious problems such as increasing shortage, pollution and waste (Boursianis et al., 2021; Duque et al., 2021; Yan et al., 2021). The rational development and utilization of water resources can be realized by building water storage projects (such as reservoirs) and inter basin water transfer projects according to local conditions, and managing the overall operation of these water conservancy projects (Shiau et al., 2021). Reservoir operation refers to the regulation of natural runoff by utilizing the reservoir’s reserving capacity (Zio and Bazzo, 2011; Ji and Wei, 2022; Zhang et al., 2022). The rational use of reservoir operation can not only reduce and relieve flood, but also store flood and make up for drought, improve utilization efficiency of water resources, etc.

Reservoir operation scheme is to generate a set of feasible operation schemes based on the results of real-time hydrological forecast and on the premise of determining the operation objectives and constraints (Wu et al., 2022a). Decision-making for reservoir operation scheme is to select the advantages and disadvantages of several feasible operation schemes at the future time of the reservoir (Zhu et al., 2017a; Vassoney et al., 2021), so as to make the reservoir obtain the maximum benefit of flood control and benefit.

The evaluation of complex system schemes often involves many indicators, and the relationship between indicators is also complex. Conventional scheme decision-making methods mainly adopt the combination of qualitative and quantitative methods, and the fusion of objective information and subjective information, such as fuzzy optimization method (Xu and Zhao, 2008; Ignatius et al., 2018; Sitorus and Brito-Parada, 2022), grey relational analysis (GRA) (Xia et al., 2016; Tian et al., 2018; Cai et al., 2021), TOPSIS method (Liu and Zhang, 2014; Imam and Gurol, 2018), projection pursuit (PP) (Lan and Huang, 2018; Lee, 2018; Cho and Lee, 2021), analytic hierarchy process (AHP) (Wang et al., 2021; Yu et al., 2021; Ye and Chen, 2022) and artificial neural network (Galdo et al., 2021; Yuan et al., 2021; Leng and Huang, 2022). In the decision-making field of reservoir operation schemes, Zhu et al. (2017b) used TOPSIS method, fuzzy optimization method and fuzzy matter-element method to rank all feasible flood control alternatives of multi-reservoir system, and the optimization scheme provides support for decision-making. Yang et al. (2021) proposed a solution framework for multi-attribute decision-making of cascade reservoirs under multiple uncertainties, and adopted improved SMAA-GCA&TOPSIS for stochastic decision-making. Wang et al. (2020) introduced the concept of subjective trade-off rate (STOR) to measure the preference of decision-makers for each target, and combined with ecological risk analysis to select the most appropriate operation rules for the Three Gorges Reservoir.

The conventional scheme decision-making method generally has problems such as large computation, low efficiency, subjectivity, dimension disaster and poor universality, which cannot well reflect the complex relationship between the evaluation object and the evaluation index (Jain et al., 2021). With the development of artificial intelligence (Hassabis et al., 2017; Wu et al., 2022b), deep learning (Janiesch et al., 2021) and big data (Ying., 2014), many scholars began to try to apply intelligent methods to the research of scheme selection. In order to promote the research of intelligent decision-making in joint operations, Hu et al. (2020) proposed a method of air attack operation scheme selection based on neural network. Compared with the traditional decision-making method, the scheme selection effect of this model is better; Chen (2021) proposed a teaching quality scheme decision-making model based on information fusion and optimized RBF neural network decision algorithm; Based on big data and neural network technology, Fang et al. (2021) proposed a novel method for the selection of football tactical command scheme, which achieved good experimental results and provided a new idea for the combination of football and computer science.

Since the neural network has the advantages of high computational efficiency and strong nonlinear fitting ability (Schmidhuber, 2015; Dong et al., 2021; Shi et al., 2022), it can well reflect the nonlinear characteristic relationship in the process of scheme decision making, so as to be closer to the real scheme selection actual scene. Fabianowski et al. (2021) developed a neural network model for the decision-making of bridge management, and ranked the alternatives. Compared with the hybrid algorithm of Extent Analysis Fuzzy Analytic Hierarchy Process (EAFAHP) and Dominant Analytic Hierarchy Process (DAHP), the high accuracy of the neural network was verified. Abdelrasoul et al. (2022) used a cascade forward backpropagation neural network to select the appropriate mining method, and compared it with a variety of multi-criteria decision-making methods, discussed its applicability, subjectivity, qualitative and quantitative data, sensitivity and effectiveness. The experimental results show that CFBPNN is easier to apply and more accurate than traditional tools. Wang and Tian (2018) designed the genetic algorithm ANN to optimize the connection weight and threshold in the optimal BP network, and established the nonlinear relationship between the mining method of thin coal face and geological conditions. However, due to the small sample size, the neural network established in this study needs to be improved regularly. The biggest difficulty in the application of neural network is related to the process of network learning. The neural network adjusts the network parameters in an iterative way to reduce the root mean square error. Using different input combinations to determine the optimal network architecture can improve network performance, which requires a large number of highly diverse input data.

After studying and analyzing the characteristics of reservoir operation scheme data, convolutional neural network (Gu et al., 2018), generative adversarial network (Goodfellow et al., 2020) and whale optimization algorithm (Mirjalili and Lewis, 2016) are applied to the research of decision-making method of reservoir operation scheme. In order to solve the problem that the data of reservoir operation scheme is few and some evaluation index data are lack of characteristics, the data augmentation algorithm IWGAN is proposed. IWGAN combines Wasserstein distance, gradient penalty and loss function of difference based on GAN, and dynamically adds random noise in the process of model training, By alternately training generator and discriminator of IWGAN on the existing data set, the characteristics and laws of reservoir operation scheme data are continuously learned to generate high-quality data for expansion. The whale optimization algorithm is improved by introducing the initial population of Logistic chaotic map, nonlinear convergence factor, adaptive weight and Levy flight disturbance strategy. The improved whale optimization algorithm IWOA is used to optimize the CNN hyperparameters of the reservoir operation scheme decision-making model. Finally, the experiment verifies that IWGAN has better data augmentation effect, IWOA has higher convergence accuracy, stronger search ability, better stability, and the decision-making model IWGAN-IWOA-CNN of reservoir operation scheme has higher prediction accuracy, the scheme it selects has good reliability.

## 2 Methods

### 2.1 Convolutional neural network

CNN is a feedforward neural network with deep structure and translation invariance inspired by biology (Gu et al., 2018). The basic structure of CNN is composed of input layer, convolution layer, pooling layer, full connection layer and output layer. The convolution layer extracts feature from the input data, the pooling layer selects features and filters information from the output results of the convolution layer, and the full connection layer classifies or regresses the extracted feature expression using the activation function. Originated from the data-driven idea, convolutional neural network does not need to carry out a detailed mathematical modeling of the system. It can mine the mapping relationship between input and output by learning and training the sample data, and then can effectively predict the output.

### 2.2 An improved generative adversarial network algorithm

#### 2.2.1 Generative adversarial network

**Figure 1:**
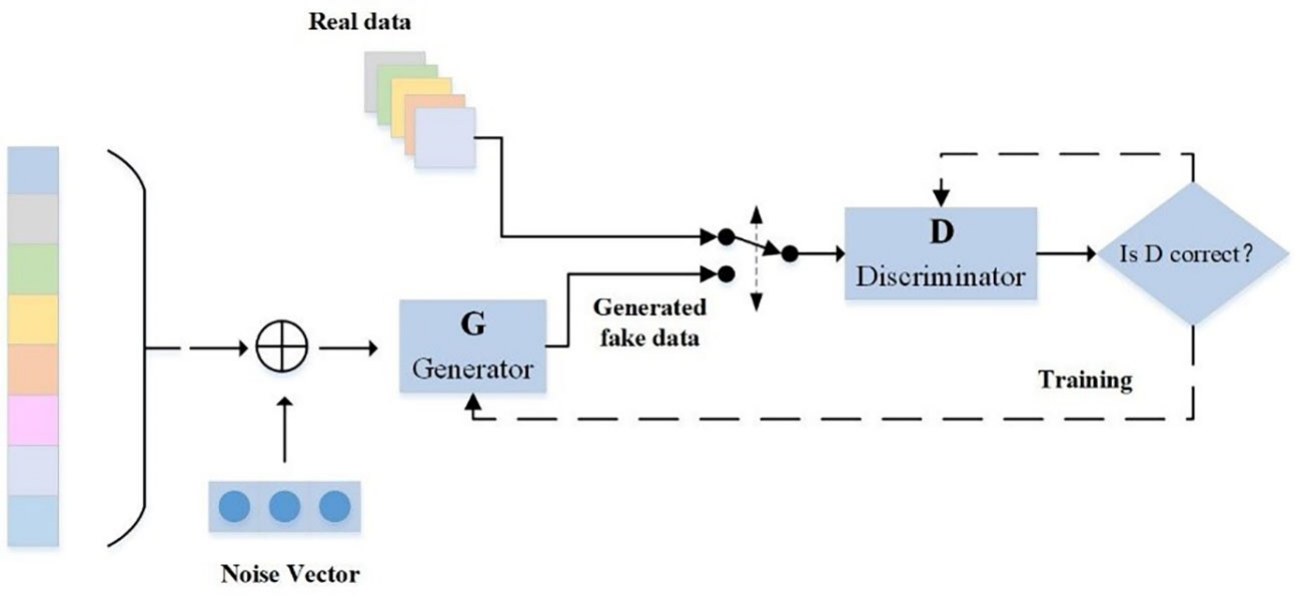
Basic structure of generative adversarial network.


GAN is usually composed of two parts: a generator and a discriminator (Goodfellow et al., 2020). The generator aims to learn the potential distribution of the real sample data and generate new samples that can be confused with the real. The discriminator aims to correctly distinguish whether the input data is from the real data or the data generated by the generator. The two eventually achieve Nash equilibrium after continuous alternating confrontation training (Wu et al., 2022c). The basic structure of GAN is shown in **Figure 1** Generator G generates virtual data G(z) from the input random noise, discriminator D randomly obtains the input from the data set fused by the real data set and the data generated by generator G, and outputs a single probability value of the sample from the real data set. During training, discriminator D should maximize the task of assigning correct labels to real data and generated data, while generator G should try to generate data similar to real data that discriminator D cannot distinguish. The loss function is:

(1) $$\begin{array}{c} min_{G}max_{D}V\left(D,G\right)=E_{x\sim P_{r}}\left[\log D\left(x\right)\right]+E_{Z\sim P_{z}}\left[\log\left(1-D\left(G\left(z\right)\right)\right)\right] \end{array}$$

Where, *x* is the real data, *z* is the input of the generator, *G*(*z*) is the generator *G* generated synthetic data, *D*(*x*) is the true probability of the discriminator *D* judging the real data, *D*(*G*(*z*)) is the true probability of judging the generated data, and *V*(*D*,*G*) represents the training process of GAN.

#### 2.2.2 A generative adversarial network based on Wasserstein distance

Arjovsky et al. (2017b) proposed a generative adversarial network based on Wasserstein distance (WGAN) to solve the problems of difficult convergence and poor controllability of GAN. WGAN uses Wasserstein distance instead of JS divergence (Huang, 2020) in GAN to measure the effective distance between real data and generated data distribution. Wasserstein distance is defined as:


(2) $$W\left(P_{r},P_{g}\right)=inf_{\delta \sim \prod^{}\left(P_{r},P_{g}\right)}E_{\left(x,y\right)\sim \delta }\left[\left|\left|x-y\right|\right|\right]$$

Where, *P*_*r*_ is the real data distribution, *P*_*g*_ is the generated data distribution, ∏^​^(*P*_*r*_,*P*_*g*_) is the set of joint probability distributions of *P*_*r*_ and *P*_*r*_, (*x*,*y*)~*δ* represents sampling a group of samples (*x*,*y*) from the joint distribution *δ*, and calculates the distance of the pair of samples, and then calculates the expectation *E*_(*x*,*y*)~*δ*_[||*x*−*y*||]. *W*(*P*_*r*_,*P*_*g*_) is the expectation infimum of the joint probability distribution *δ*(*x*,*y*). The smaller *W*(*P*_*r*_,*P*_*g*_) is, the higher the similarity between the real data distribution and the generated data distribution. The loss function of WGAN is:


(3) $$\begin{array}{c} L=min_{G}max_{D\in Lip_{1}}E_{x\sim P_{r}}\left[\left(D\left(x\right)\right)\right]+E_{x\sim P_{g}}\left[1-\left(D\left(x\right)\right)\right] \end{array}$$

where, *D*∈*Lip*_1_ means that the discriminator meets the 1-lipschitz continuity condition.

#### 2.2.3 An improved WGAN algorithm

In order to ensure the Lipschitz continuity condition of the discriminator, WGAN checks whether all parameters exceed a certain range [−*c*,*c*] every time the parameters *ω* of the discriminator are updated. If beyond this range, set the parameter greater than *c* as *c* and the parameter less than -*c* as -*c*. This weight clipping strategy (Han et al., 2020) will lead to changes in the structure of the parameter matrix of the discriminator network and the corresponding relationship between the parameters, The extreme phenomenon of maximum or minimum value of parameters, gradient disappearance and gradient explosion occur (Goodfellow, 2016; Gulrajani et al., 2017; Arjovsky and Bottou, 2017a). To solve these problems, this paper proposes an improved WGAN algorithm IWGAN, which adds the gradient penalty term and the finite difference in the loss function, and introduces the dynamic random noise adjustment algorithm.

WGAN restricts Lipschitz continuity condition in the whole sample space, while IWGAN only imposes gradient penalty constraint on the area between the real sample data and the generated sample data. The L2 norm of the gradient is constrained near 1 on the optimal path from the generated distribution to the real distribution. Based on the idea of random interpolation and bilateral penalty, a gradient penalty term is designed:


(4) $$\begin{array}{c} GP{|}_{x}≔E_{x}{\left[{\left|\left|\nabla_{x}D\left(x\right)\right|\right|}_{2}-1\right]}^{2} \end{array}$$

where, $\hat{x}=\in x+\left(1-\in \right)y$ , , *x*∈*P*_*r*_, *y*∈*P*_*g*_.

The loss function of adding gradient penalty term is updated as:


(5) $$\begin{array}{c} L=min_{G}max_{D\in Lip_{1}}E_{x\sim P_{r}}\left[\left(D\left(x\right)\right)\right]+E_{x\sim P_{g}}\left[1-\left(D\left(x\right)\right)\right]\\ +\lambda E_{x\sim P_{penalty}}{\left[{\left|\left|\nabla_{x}D\left(x\right)\right|\right|}_{2}-1\right]}^{2} \end{array}$$

where, *λE*_*x*~*penalty*_[||∇_*x*_ *D*(*x*)||_2_−1]^2^ is a penalty term, whose purpose is to make smooth to accelerate the convergence speed of the model, means that random sampling (also known as penalty term sampling) is carried out between the real data distribution *P*_*r*_ and the generated data distribution *P*_*g*_, and ∇_*x*_ *D*(*x*) means to calculate the derivative of *x*.

Considering that the gradient penalty is weak for the continuity constraint in a small range on the Lipschitz continuity condition, and even discontinuous in some extreme cases, the difference item is added to the loss function to accelerate the convergence of the model by enhancing the continuity constraint of the gradient penalty, so as to improve the stability of network training.

Suppose the discriminator *f*_*w*_(*x*) is differentiable on Kantorovich-Rubinstein, , *x*_2_~*p*_*x*_*g*__, *l*∈*Uniform*[0,1], and randomly interpolates between *x*_1_ and *x*_2_, with *x*_*l*_=(1−*l*)*x*_1_+*lx*_2_, meet ||*f* ^*^(*x*_*l*1_)−*f* ^*^(*x*_*l*2_)||=||*x*_*l*1_−*x*_*l*2_||, which satisfies the Lipschitz continuity condition. At this point, *x*_*l*_ satisfies the distribution *p*_*x*_*l*__.

The gradient constraint items incorporating the idea of difference are as follows:


(6) $$\begin{array}{c} DGP{|}_{x_{l1},x_{l2}}≔E{\left[\frac{\left|\left|D\left(x_{l1}\right)-D\left(x_{l2}\right)\right|\right|}{\left|\left|x_{l1}-x_{l2}\right|\right|}-1\right]}^{2} \end{array}$$

The loss function of difference item is added as follows:

Where, , *γ*_2_, *γ*_3_ are super parameters.

In the process of model training, some Gaussian noise conforming to the distribution is added to each layer of generator network and input layer of discriminator network, and the noise size is dynamically adjusted. The algorithm is shown in **Algorithm 1**. At the initial stage of model training, the distance between the generated data distribution and the real data distribution is far, and adding large noise will not affect the convergence speed of the model. As the training depth of the model deepens, adding too much noise may cause the model parameters to oscillate near the Nash equilibrium point. Therefore, with the increase of the number of model training, the noise scale should be gradually reduced, so as to accelerate the convergence speed of the model. The flow chart of IWGAN is given in **Appendix A**.

**ALGORITHM 1.** Dynamic random noise adjustment algorithm

**Input:**Initial noise level *σ* _0_ ;Number of iterations *T*; Attenuation rate *μ* ;Threshold *k* ; **Output:**Current noise *σ* _ *c*+1_ ; 1: **for** epoch =1 to **do** 2: $\sigma_{c+1}=\sigma_{c}-\sigma_{0}^{-1}\left(1+\mu T\right)$ 3: **if** *σ*_*c*_≥*k* **then** 4: $\xi \sim N\left(0,\sigma_{c}^{2}\right)$ 5: **else**6: $\xi \sim N\left(0,\sigma_{k}^{2}\right)$ 7:**end for**

### 2.3 An improved whale optimization algorithm

Whale optimization algorithm (Mirjalili and Lewis, 2016) (WOA) simulates the predatory behavior of humpback whales, and searches for the optimal solution of the problem through three strategies: shrinking and surrounding prey, spiral bubble net predation and random foraging. In this paper, an improved whale optimization algorithm (IWOA) is proposed to solve the problems of slow convergence speed, difficulty in coordinating global and local search ability, and easy to fall into local optimization of WOA (Al-Zoubi et al., 2018; Sun et al., 2018). **Algorithm 2** shows the IWOA.

**ALGORITHM 2.** Improved whale optimization algorithm (IWOA)

**Input:**Population size N, Spatial dimension Dim, The maximum number of iterations T **Output:**Optimal solution X^*^ 1.Initialize the whale population using the Logistic chaotic mapping method *X*_*i* _(*i*=1,2,…,*n*) 2.Calculate the fitness of each search agent 3. X^*^ is the best search agent 4.If the optimal fitness value remains unchanged for 5 consecutive generations, use Eq. (15) to update the position of Levy flight disturbance 5.**while** t<T **do** 6. **for** i=1 to N **do** 7. Update A, D, p and 8. **if1** (p<0.5) 9. **if2** (|A|≥1) 10. Select a random search agent *X*_*rand* _ 11. Update the position of the current search agent by the Eq. (13) 12. **else if2** (|A|<1) 13. Update the position of the current search agent by the Eq. (12) 14. **end if2** 15. **else if1** (p p≥0.5) 16. Update the position of the current search agent by the Eq. (11) 17. **end if1** 18. **end for** 19. Check if any search agent goes beyond the search space and amend it 20. Calculate the fitness of each search agent 21. Update X^*^ if there is a better solution 22. t=t+1 23.**end while**

Firstly, the chaotic map (Prasad et al., 2021) is used to initialize the whale population, so that the initial position sequence is evenly distributed in the search space, which effectively improves the convergence accuracy and stability of the algorithm. The Logistic chaotic mapping method is used to initialize the whale population. The mathematical expression is as follows:


(8) $$\begin{array}{c} x_{n+1}=\mu x_{n}\left(1-x_{n}\right) \end{array}$$

where, *x*_*n*_ is the state quantity and *μ* is the logistic parameter.

The nonlinear convergence factor $\vec{a}$ is designed to decrease slowly in the early iteration process, making the value of the parameter *A* larger, so as to improve the global search ability; In the later iteration process, it decreases rapidly, making the value *A* smaller, so as to improve the local search ability. The formula is as follows:


(9) $$\begin{array}{c} \vec{a}=\{\begin{array}{cc} 2-e\times {\left(\frac{t-\cos\left(\pi \times \frac{t}{T}\right)}{T-1}\right)}^{2} & t\leq 0.5T\\ 2^{\frac{T-t\times \cos\left(\pi \times \frac{t-0.5T}{T}\right)}{T-1}} & t>0.5T \end{array} \end{array}$$

where, *T* is the maximum number of iterations and *t* is the current number of iterations. Considering that the convergence speed of WOA is slow at the late stage of iteration, and due to the fixed weight during local search, WOA will oscillate around the current optimal solution, resulting in falling into local optimum. Therefore, it is hoped that WOA can appropriately expand the global search scope and enhance the ability to jump out of the local optimum while retaining the local exploration ability at the end of the iteration. Therefore, an adaptive weight *ω* is designed, and the formula is as follows:


(10) $$\begin{array}{c} \omega =\frac{4}{\pi }\times \tan\left(\frac{e^{\frac{t}{T}-1}}{e-1}\right) \end{array}$$

The location update formula of IWOA is as follows:


(11) $$\begin{array}{c} \vec{X}\left(t+1\right)=\vec{D^{\prime }}\cdot e^{bl}\cdot \cos\left(2\pi l\right)+\left(1-\omega \left(t\right)\right)\times \vec{X^{*}}\left(t\right)\;\;\;\;\;\;\;\;p\geq 0.5 \end{array}$$

(12) $$\begin{array}{c} \vec{X}\left(t+1\right)=\omega \left(t\right)\times \vec{X^{*}}\left(t\right)-\vec{A}\cdot \vec{D}\;\;\;\;\;\;\;\;p<0.5\cup^{}A<1 \end{array}$$

(13) $$\begin{array}{c} \vec{X}\left(t+1\right)=\;\omega \left(t\right)\times \vec{X_{rand}}\left(t\right)-\vec{A}\cdot {\vec{D}}_{rand}\;\;\;\;\;\;\;\;p<0.5\cup^{}A\geq 1 \end{array}$$

The Levy flight disturbance mechanism is introduced (Deepa and Venkataraman, 2021), and the disturbance is added to the position update mode to make the algorithm not easy to fall into local optimization and premature convergence. The location update formula is as follows:


(14) $$\begin{array}{c} {\text{X}}^{l}\left(t\right)=\text{X}\left(t\right)+\alpha ⊕\text{Levy}\left(\lambda \right) \end{array}$$

Where, *X* ^*l*^(*t*) is the position after adding Levy flight disturbance, *α* is the step size factor that changes dynamically with the number of iterations, ⊕ is the point multiplication, and Levy(*λ*) represents the Levy distribution that obeys the *λ*, and the formula is as follows:


(15) $$\begin{array}{c} \text{Levy}∼u=t^{-\lambda }\;\;\;\;\;\;\;\;1<\lambda \leq 3 \end{array}$$

(16) $$\begin{array}{c} \alpha \left(t\right)=\frac{t}{T}\sin\left(1-\frac{t}{T}\right)\cdot r \end{array}$$

Where, *t* is the current number of iterations, *T* is the maximum number of iterations, and is the adjustment factor. The Levy distribution is approximately simulated by Mantegna algorithm (Mantegna, 1994), and the formula is as follows:


(17) $$\begin{array}{c} s=\frac{\mu }{|\nu {|}^{\frac{1}{\beta }}} \end{array}$$

Where, , *μ* and obey the normal distribution of the $\sigma_{\mu }^{2}$ and $\sigma_{\nu }^{2}$ . In order to ensure that the new position after disturbance is better than the original position, the greedy selection strategy is used to compare the fitness of the two to retain the new position with better fitness. The formula is as follows:


(18) $$\begin{array}{c} x_{i}\left(t\right)=\{\begin{array}{c} x_{i}\left(t\right),\;\;fit\left(x_{i}^{'}\left(t\right)\right)<fit\left(x_{i}\left(t\right)\right)\\ x_{i}^{'}\left(t\right),\;\;fit\left(x_{i}^{'}\left(t\right)\right)\geq fit\left(x_{i}\left(t\right)\right) \end{array} \end{array}$$

### 2.4 Overall research methodology

This paper refers to Hu et al. (2021), uses CNN as the benchmark model for reservoir operation scheme selection, and uses fuzzy optimization theory to construct CNN training samples. Firstly, the evaluation index system is constructed according to the fuzzy optimization theory; Secondly, the weight of each evaluation index is determined, the subjective weight is determined by analytic hierarchy process, the objective weight is determined by entropy weight method, and the comprehensive weight is obtained by coupling the subjective and objective weights by game theory; Finally, the comprehensive evaluation value of the scheme is calculated by fuzzy comprehensive evaluation, and the initial sample of CNN model is obtained. Due to the lack of data of the original reservoir operation scheme, IWGAN proposed in this paper is used to augment the data set of the original reservoir operation scheme, and improve the prediction accuracy of the model and the reliability of scheme selection. IWOA is used to optimize the parameters of CNN model and determine the optimal network structure, the specific process is given in **Appendix B**.

**Figure 2:**
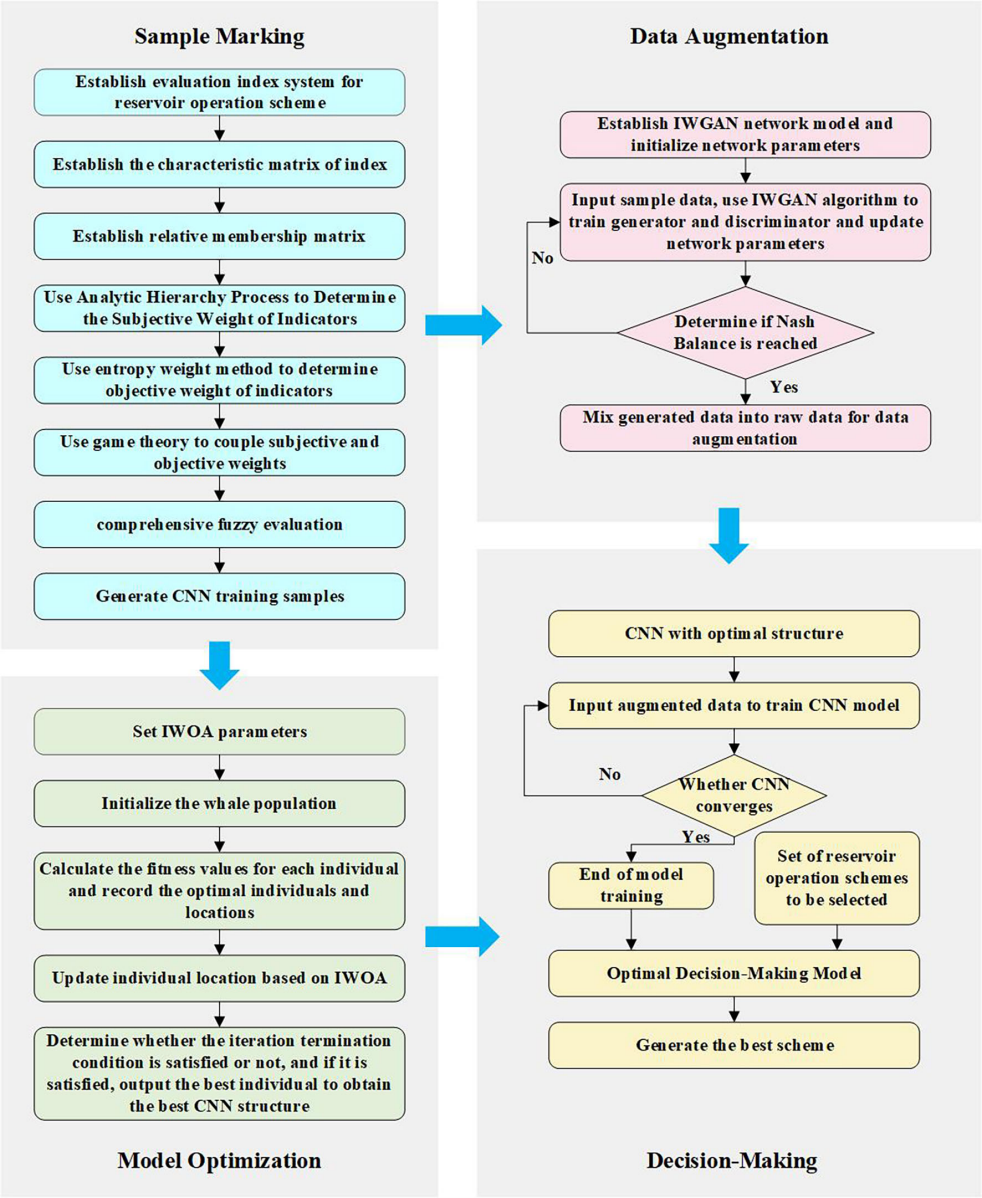
The framework of the decision-making method for reservoir operation schemes.

The evaluation index and comprehensive evaluation value in the sample data are respectively used as the input and output of CNN model. The comprehensive evaluation value of the scheme is predicted by CNN model, so as to evaluate the advantages and disadvantages of the scheme. According to the principle of maximum membership, the scheme with the maximum comprehensive evaluation value is the optimal scheme. The overall flow chart is shown in **Figure 2**.

### 2.5 Data preprocessing

In this paper, the Xidayang reservoir (Hu et al., 2021) is selected as the research object, and many reservoir operation schemes generated by the reservoir multi-objective operation model are used as the experimental data set, including the flood data of the Xidayang reservoir with 10-year return period (data set 1), 20-year return period (data set 2) and 50-year return period (data set 3). Each data has six characteristic columns, which are water resources conversion, water level recovery level, peak shaving amplitude, drawdown depth, recovery time and maximum water level variation. The experimental data were randomly divided into training set, verification set and test set according to the ratio of 6:2:2.

Data preprocessing includes: (і) missing value processing, select the value of adjacent pre discharge time under the same pre discharge flow to fill in the missing value; (ii) normalization processing, the evaluation index involves two types of indicators, benefit type and cost type, and the normalization methods are (19) and (20) respectively; and (iii) denoising processing, the 3 *σ* principles of normal distribution and kernel smoothing method (Ahmad et al., 2001) are selected for data denoising.


(19) $$\begin{array}{c} r_{ij}=\frac{x_{ij}-x_{jmin}}{x_{jmax}-x_{jmin}}\;\;\;i\in \left[1,m\right]\;\;j\in \left[1,n\right] \end{array}$$

(20) $$r_{ij}=\frac{x_{jmin}-x_{ij}}{x_{jmax}-x_{jmin}}\;\;\;i\in \left[1,m\right]\;\;j\in \left[1,n\right]$$

where, *x*_*ij*_(*i*=1,2,…,*m*;*j*=1,2,…,*n*) is the eigenvalue of the scheme evaluation index, *n* is the number of schemes to be optimized, and is the number of evaluation indexes.

## 3 Results and discussion

In order to verify the effectiveness of the IWGAN-IWOA-CNN model proposed in this paper, three experiments are carried out. The first part of the experiment verifies the data augmentation effect of IWGAN, the second part of the experiment verifies the optimization performance of IWOA, and the last part of the experiment compares this model with other scheme decision-making models, so as to verify the superiority of the model proposed in this paper.

### 3.1 Data augmentation analysis of generative adversarial network

In order to verify the data augmentation effect of IWGAN (the network structure of generator and discriminator is given in **Appendix C**, and the training process of IWGAN is given in **Appendix D**), the experimental data set is input into IWGAN model for confrontation game training. The experiment uses Adam optimizer, and sets the learning rate as 0.00001, *β*_1_ =0.9, *β*_2_ =0.99, the batch size as 64, and the epoch as 10000.

**Figure 3:**
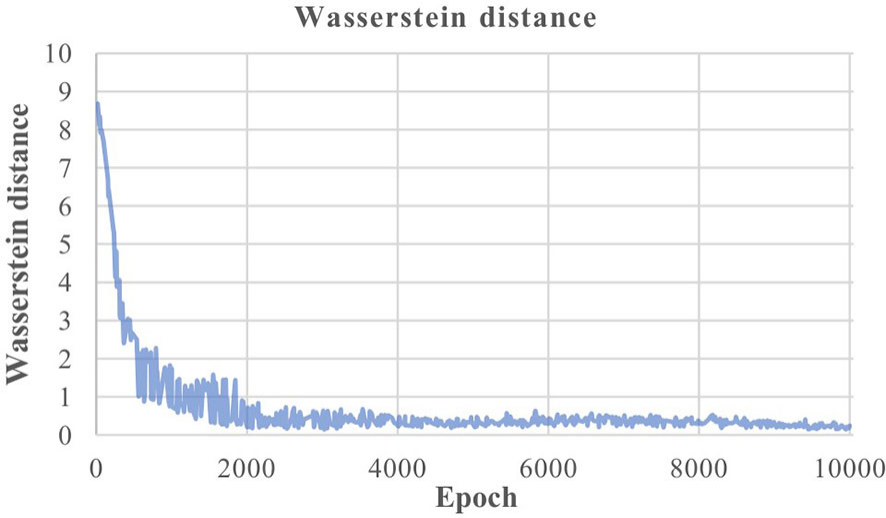
Wasserstein distance change in dataset 1.

Taking dataset 1 as an example, **Figure 3** shows the change process of Wasserstein distance during the training process. It can be seen from the figure that at the beginning of training, the Wasserstein distance value is large, and the similarity between the data generated by the generator and the real data distribution is low. With the progress of training, the Wasserstein distance gradually decreases, indicating that the generated data distribution gradually approaches the real data distribution. When the number of training times reaches about 4000, the Wasserstein distance approaches zero and fluctuates around it, indicating that the generator and the discriminator reach Nash equilibrium.

**Figure 4:**
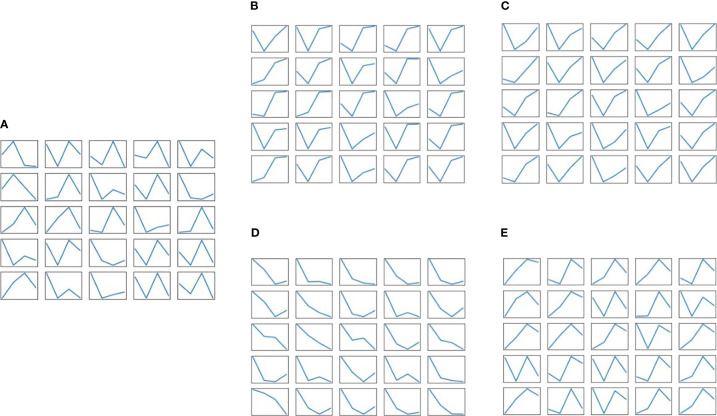
The real sample data and the generated sample data are randomly sampled, and the generated sample data are saved every 50 epochs. **(A)** Real sample data. **(B)** Generated sample data when e=50. **(C)** Generated sample data when e=1000. **(D)** Generated sample data when e=2850. **(E)** Generated sample data when e=4000.

At the same time, in order to reflect the dynamic learning process of IWGAN model, the real sample data and the generated sample data are randomly sampled, and the generated sample data are saved every 50 epochs. **Figure 4A** is a visual image of real sample data distribution. **Figures 4B–E** show the generated sample data of IWGAN model when the number of iterations is 50, 1000, 2850 and 4000 respectively. It can be seen that at the initial stage of training *e* =50, the fluctuation law of the data generated by the generator is quite different from that of the real data. With the deepening of IWGAN training, the model gradually learns the change law of the real data, the distribution of the generated data and the real data is getting closer, and IWGAN is also constantly improving its data augmentation effect and the quality of the generated data. When *e* =4000, the data generated by IWGAN is basically consistent with the real data distribution. Therefore, the data generated by IWGAN has a certain representation ability, which is applicable to the enhancement of the reservoir operation scheme data in this paper.

Through the above analysis, it can also be concluded that the Wasserstein distance in the discriminator is closely related to the data quality generated by the generator. The Wasserstein distance can indicate the training process to a certain extent. The smaller the Wasserstein distance, the better the training effect of IWGAN model. When the number of iterations of the model reaches about 4000, the Wasserstein distance approaches zero and tends to be flat, which proves that the network converges and the model reaches Nash equilibrium. At this time, the generator generates high-quality sample data, and its distribution has been close to the real data distribution. Finally, this paper uses the model with 4000 iterations on dataset 1 for data augmentation.

### 3.2 Performance analysis of whale optimization algorithm

To verify the performance of IWOA, this paper compares it with the traditional whale optimization algorithm (WOA), particle swarm optimization (PSO) (Kennedy and Eberhart, 1995), cuckoo algorithm (CS) (Yang and Suash, 2009) and gray wolf algorithm (GWO) (Mirjalili et al., 2014) on 11 test functions. The test functions are shown in **Table 1**, and the test results are shown in **Table 2**. The optimization accuracy of the algorithm is the absolute value of the error between the actual optimal solution and the theoretical optimal solution. In this paper, the average (*Ave*) and standard deviation (*Std*) of the optimization accuracy are used to reflect the convergence accuracy and stability of the algorithm. The calculation formula is as follows:

**Table 1:** Benchmark functions.

Serial numb-er	Expression	Type	Dimen-sion	Search scope	Theoretical minimum
1	$f_{1}(x)=\sum_{i=2}^{n}x_{i}^{2}$	U	30	[-100,100]	0
2	$f_{3}(x)=\sum_{i=2}^{n}\left|x_{i}\right|+\prod_{i=1}^{n}\left|x_{i}\right|$	U	30	[-10,10]	0
3	$f_{3}(x)=\sum_{i=1}^{n}\left(\sum_{j=1}^{i}x_{j}\right)$	U	30	[-100,100]	0
4	$f_{4}(x)\underset{i}{\max}\left\{\left|x_{i}\right|,1\leq i\leq n\right\}$	U	30	[-100,100]	0
5	$f_{5}\left(x\right)=\sum_{i=1}^{n-1}\left[100{\left(x_{i+1}-x_{i}^{2}\right)}^{2}+{\left(x_{i}-1\right)}^{2}\right]$	U	30	[-30,30]	0
6	$f_{6}(x)=\sum_{i=1}^{n}\left[100{\left(x_{i+1}-x_{i}^{2}\right)}^{2}+{\left(x_{i}-1\right)}^{2}\right]$	N	30	[-1.28,1.28]	0
7	$f_{7}(x)=\sum_{i=1}^{n}-x_{i}\sin\left(\sqrt{\left|x_{i}\right|}\right)$	M	30	[-500,500]	-12569.5
8	$f_{8}(x)=\sum_{i=1}^{n}\left[x_{i}^{2}-10\cos(2\pi x_{i})+10\right]$	M	30	[-5.12,5.12]	0
9	$f_{9}\left(x\right)=20-20\exp\left(-0.2\sqrt{\frac{1}{n}\sum_{i=1}^{n}x_{i}^{2}}\right)$ $-\exp\left(\frac{1}{n}\sum_{i=1}^{n}\cos\left(2\pi x_{i}\right)\right)+e$	M	30	[-32,32]	0
10	$f_{11}(x)=\frac{1}{4000}\sum_{i=1}^{n}x_{i}^{2}-\prod_{i=1}^{n}\cos\left(\frac{x_{i}}{\sqrt{i}}\right)+1$	M	30	[-600,600]	0
11	$\begin{array}{c} f_{11}(x)=0.1\{\sin^{2}\left(3\pi x_{1}\right)+\sum_{i=1}^{n}{\left(x_{i}-1\right)}^{2}\left[1+\sin^{2}\left(33\pi x_{1}\right)+1\right]\\ +{\left(x_{n}-1\right)}^{2}\left[1+\sin^{2}\left(2\pi x_{n}\right)\right]\}+\sum_{i=1}^{n}\mu \left(x_{i}5,100,4\right) \end{array}$	M	30	[-50,50]	0

U is a unimodal function, which means that there is only one strictly local maximum (peak) real value function in the interval under consideration.

M is a multimodal function, which means a real-valued function with multiple local maximums (peaks) in the interval under consideration.

**Table 2:** Experimental results of different optimization algorithms.

Function	Index	PSO	GWO	CS	WOA	IWOA
*f*_1_	Ave	2.75E-74	1.10E-172	9.24E-148	2.21E-225	**0**
	Std	1.01E-61	2.54E-168	7.91E-134	2.25E-193	**0**
*f*_2_	Ave	5.5E-121	2.87E-201	7.73E-50	4.50E-186	**4.92E-341**
	Std	2.90E-116	2.36E-197	4.69E-49	1.51E-174	**1.36E-340**
*f*_3_	Ave	3687.4967	1.58E-276	1965.6028	1.79E-74	**0**
	Std	1435.1613	5.16E-276	2330.0190	**0**	**0**
*f*_4_	Ave	5.69E-109	1.77E-185	1.08E-12	1.42E-99	**1.66E-280**
	Std	5.04E-107	1.34E-184	1.96E-12	**0**	**0**
	Ave	8.18E+01	2.17E-01	5.78E+01	4.19E-01	**6.58E-02**
	Std	**1.27E-03**	9.50E-01	1.91E+01	2.62E-01	1.85E-02
*f*_6_	Ave	9.66E-01	4.32E-02	7.13E-01	2.86E-03	**1.66E-04**
	Std	1.38E-02	2.85E-02	8.12E-01	3.67E-03	**2.46E-04**
*f*_7_	Ave	-2.23E+01	-3.49E+02	-5.33E+03	-2.18E+04	**-1.56E+04**
	Std	5.28E+01	1.28E+03	6.60E+02	1.32E+03	**2.27E+00**
*f*_8_	Ave	**0**	**0**	4.2E-17	**0**	**0**
	Std	**0**	**0**	4.01E-16	**0**	**0**
*f*_9_	Ave	2.31E-07	2.27E-01	7.25E-04	1.54E-12	**7.54E-16**
	Std	8.31E-06	1.21E-01	3.65E-04	7.46E-10	**2.47E-32**
*f*_10_	Ave	6.97E-13	**0**	1.15E-17	1.51E-35	**0**
	Std	1.69E-13	1.17E-16	1.83E-18	1.32E-34	**0**
*f*_11_	Ave	5.09E-84	4.41E-88	1.23E-52	9.02E-82	**0**
	Std	5.58E-67	1.27E-78	4.63E-53	1.12E-81	**0**

Bold values indicate the best performance for this item.


(21) $$Ave=\frac{\sum_{i=1}^{N}\left|\left|f\left(X^{*}\right)-f\left(X_{opt}\right)\right|\right|}{N}$$

(22) $$Std=\sqrt{\frac{\sum_{i=1}^{N}{\left(f\left(X^{*}\right)-Ave\right)}^{2}}{N}}$$

Where, *X*_*opt*_ is the theoretical optimal solution, and *N* is the total number of experiments.

**Figure 5:**
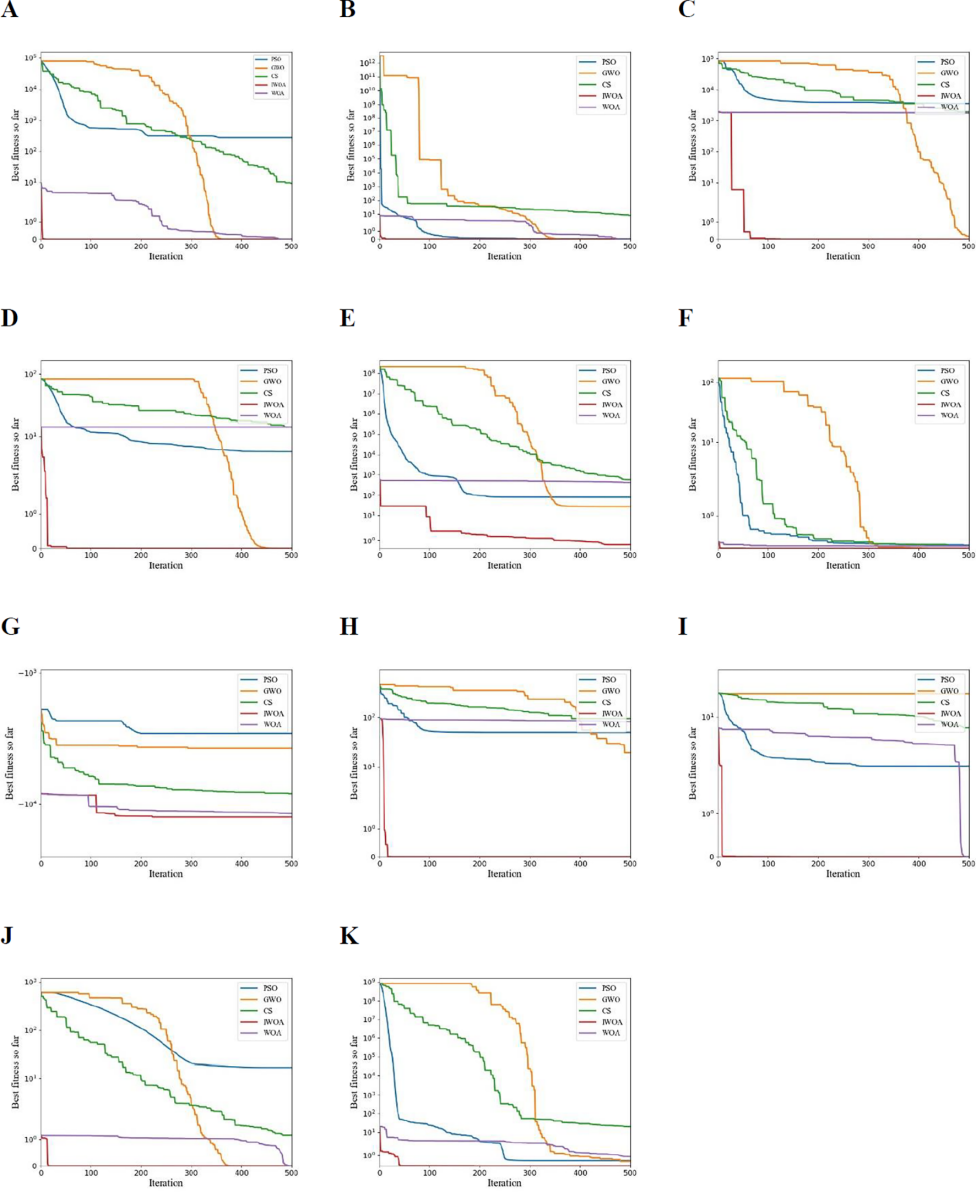
Convergence curves of different optimization algorithms on 11 test functions, **(A–K)** correspond to *f*_1_~*f*_11_ respectively.

**Figure 5** shows the convergence curves of the above five optimization algorithms on 11 test functions. The experiment set the population size of all algorithms *N* =30, the maximum number of iterations *T* =500, and each algorithm ran independently for 30 times. Set learning factor *c*_1_ = *c*_2_ =2, inertia weight *w*_*min*_ =0.1, *w*_*max*_ =0.4 in PSO; Set parameters in CS *pa* =0.25, Levy flight parameters =0.01, *β* =1.5.

It can be seen from **Table 2** that IWOA performs better than the other four algorithms in terms of optimization effect. From the perspective of *Ave*, IWOA is better on 11 test functions. This shows that the convergence accuracy of IWOA is higher, and the optimization result of IWOA is closer to the theoretical minimum of the test function. From the perspective of *Std*, in addition to the slightly worse stability of IWOA in the function than PSO, the stability of IWOA in other test functions is better than the other four algorithms, which shows that the optimization stability of IWOA is better.

As can be seen from **Figure 5**, compared with other algorithms, the convergence curve of IWOA decreases the fastest, and on the 11 test functions, the optimal fitness convergence curve of IWOA is located below the curves of the other four algorithms, indicating that IWOA has faster convergence speed and higher convergence accuracy. It can be seen from the convergence curves of functions , and *f*_8_~*f*_11_ that the convergence curve of IWOA can be approximately regarded as a straight line. This is due to the introduction of Logistic chaotic map initialization population, nonlinear convergence factor and adaptive weight, so that the algorithm can find the optimal solution after less iterations. It can also be seen from the convergence curve of function and *f*_7_ that there are many inflection points in the convergence curve of IWOA, and the curve is abrupt, which shows that IWOA has stronger ability to jump out of local optimum. To sum up, the IWOA proposed in this paper has faster convergence speed, stronger optimization ability and better stability.

### 3.3 Effect analysis of decision-making model for reservoir operation scheme

In this paper, IWGAN is used to augment the data set of the original reservoir operation scheme, and IWOA is used to optimize the network structure of CNN and determine the optimal structure of the model (see **Appendix E**). In order to verify the effectiveness and prediction effect of IWGAN-IWOA-CNN reservoir operation scheme decision-making model, this paper conducts comparative experiments from three aspects: different data augmentation algorithms, different optimization algorithms and different scheme decision-making models.

#### 3.3.1 Comparison of different data augmentation algorithms

In order to verify the superiority of IWGAN over other data augmentation algorithms, this section compares IWGAN-IWOA-CNN model with IWOA-CNN (without data augmentation algorithm), WGAN-IWOA-CNN, GAN-IWOA-CNN and SMOTE (Douzas and Bacao, 2019) -IWOA-CNN models. **Table 3** shows the experimental results of models under different data augmentation algorithms. According to the **Table 3**, different data augmentation algorithms have improved the prediction accuracy of the model to a certain extent, but the IWGAN-CNN model proposed in this paper has better prediction effect. Compared with CNN, SMOTE-CNN, GAN-CNN and WGAN-CNN models, the MAE decreased by 29%, 27.5%, 14.4% and 13.2%, respectively; RMSE decreased by 15.2%, 23.2%, 5% and 9.7% respectively; R2 increased by 2.5%, 6.1%, 1.4% and 0.4% respectively. On the whole, IWGAN has the most significant effect on data augmentation, IWGAN-CNN model has the best robustness and generalization ability, and the effect of scheme selection is the best, followed by WGAN-CNN model. Because the loss function of GAN discriminator is defined based on JS divergence, it is difficult to solve the problems of unstable training and mode collapse, so the performance of GAN-CNN model is slightly poor. Smote algorithm is essentially an improvement on the oversampling algorithm. It is difficult to learn the distribution law of real data through its own learning ability, and it does not have the increase of effective information. Moreover, due to the randomness of oversampling, the performance of the model is greatly different and the stability is poor, so SMOTE-CNN model performs worst.

**Table 3:** Comparison of experimental results of models under different data augmentation algorithms.

Data set	Data augmentation algorithm	Evaluating indicator		
		MAE	RMSE	R^2^
Dataset 1	Raw dataset	0.0083	0.0099	0.9461
	SMOTE	0.0079	0.0097	0.9472
	GAN	0.0069	0.0088	0.9544
	WGAN	0.0068	0.0093	0.9606
	IWGAN	**0.0059**	**0.0084**	**0.9636**
Dataset 2	Raw dataset	0.0099	0.0133	0.9018
	SMOTE	0.0120	0.0151	0.8711
	GAN	0.0097	0.0119	0.9118
	WGAN	0.0088	0.0122	0.9218
	IWGAN	**0.0087**	**0.0116**	**0.9247**
Dataset 3	Raw dataset	0.0080	0.0106	0.9373
	SMOTE	0.0079	0.0104	0.9378
	GAN	0.0078	0.0102	0.9381
	WGAN	0.0079	0.0102	**0.9441**
	IWGAN	**0.0069**	**0.0101**	**0.9441**

Bold values indicate the best performance for this item.

#### 3.3.2 Comparison of different optimization algorithms

The IWGAN-IWOA-CNN model is compared with IWGAN-WOA-CNN, IWGAN-PSO-CNN, IWGAN-CS-CNN and IWGAN-GWO-CNN models to verify the effectiveness of IWGAN-IWOA-CNN model. The experimental results are shown in **Table 4**. On the whole, except that the MAE value of IWGAN-GWO-CNN model on dataset 1 is relatively minimum, in other cases, the Mae and RMSE values of IWGAN-IWOA-CNN model are relatively minimum, R2 is the closest to 1, and the model performs best. This shows that IWOA has higher optimization accuracy and better optimization effect. IWOA-CNN algorithm can effectively help CNN find the best network parameters, and IWGAN-IWOA-CNN model has higher prediction accuracy, better generalization ability and robustness

**Table 4:** Comparison of experimental results of models under different optimization algorithms.

Data set	Scheme optimization model	Evaluating indicator		
		MAE	RMSE	R^2^
Dataset 1	IWGAN-CNN	0.0056	0.0084	0.9636
	IWGAN-PSO-CNN	0.0041	0.0056	0.9808
	IWGAN-CS-CNN	0.0057	0.0069	0.9766
	IWGAN GWO CNN	**0.0030**	0.0045	0.9896
	IWGAN-WOA-CNN	0.0038	0.0052	0.9826
	IWGAN-IWOA-CNN	0.0031	**0.0040**	**0.9909**
Dataset 2	IWGAN-CNN	0.0087	0.0116	0.9247
	IWGAN-PSO-CNN	0.0085	0.0102	0.9397
	IWGAN-CS-CNN	0.0074	0.0107	0.9350
	IWGAN GWO CNN	0.0075	0.0109	0.9322
	IWGAN-WOA-CNN	0.0072	0.0103	0.9413
	IWGAN-IWOA-CNN	**0.0067**	**0.0099**	**0.9532**
Dataset 3	IWGAN-CNN	0.0069	0.0101	0.9441
	IWGAN-PSO-CNN	0.0052	0.0062	0.9808
	IWGAN-CS-CNN	0.0071	0.0090	0.9590
	IWGAN GWO CNN	0.0049	0.0069	0.9743
	IWGAN-WOA-CNN	0.0062	0.0078	0.9652
	IWGAN-IWOA-CNN	**0.0024**	**0.0032**	**0.9947**

Bold values indicate the best performance for this item.

#### 3.3.3 Comparison of decision-making models of different schemes

IWGAN-IWOA-CNN model is compared with IWGAN-IWOA-BP, IWGAN-IWOA-SVR and IWGAN-IWOA-MLP models. The experimental results are shown in **Table 5**.

**Table 5:** Comparison of different scheme decision-making models.

Data set	Scheme decision-making model	Evaluating indicator			Training time (s)
		MAE	RMSE	R^2^	
Dataset 1	IWGAN-IWOA-BP	0.0050	0.0070	0.9734	**92**
	IWGAN-IWOA-MLP	0.0069	0.0084	0.9575	110
	IWGAN-IWOA-SVR	0.0051	0.0071	0.9707	109
	IWGAN-IWOA-CNN	**0.0031**	**0.0040**	**0.9909**	101
Dataset 2	IWGAN-IWOA-BP	0.0099	0.0129	0.9121	120
	IWGAN-IWOA-MLP	0.0101	0.0126	0.9215	102
	IWGAN-IWOA-SVR	0.0101	0.0128	0.9080	95
	IWGAN-IWOA-CNN	**0.0067**	**0.0099**	**0.9532**	**87**
Dataset 3	IWGAN-IWOA-BP	0.0085	0.0106	0.9399	108
	IWGAN-IWOA-MLP	0.0078	0.0095	0.9492	**102**
	IWGAN-IWOA-SVR	0.0053	0.0072	0.9706	123
	IWGAN-IWOA-CNN	**0.0024**	**0.0032**	**0.9947**	115

Bold values indicate the best performance for this item.

According to the analysis chart, in terms of evaluation indicators, the MAE and RMSE of IWGAN-IWOA-CNN model are the smallest and R2 is the closest to 1 in data sets 1-3. Compared with IWGAN-IWOA-BP, IWGAN-IWOA-MLP and IWGAN-IWOA-SVR models, MAE decreases by 71.8%, 69.2% and 54.7%, RMSE decreases by 69.8%, 66.3% and 55.5%, and R2 increases by 5.8%, 4.8% and 2.5%. In terms of training time, in dataset 1, the training time of IWGAN-IWOA-CNN model is slightly longer than that of IWGAN-IWOA-BP model, and in dataset 3, the training time of this model is also slightly longer than that of IWGAN-IWOA-MLP and IWGAN-IWOA-BP models. However, considering the performance of the model, a slightly longer training time can greatly improve the prediction accuracy of the model and the accuracy of scheme evaluation, so the IWGAN-IWOA-CNN model proposed in this paper has the best prediction performance and scheme selection effect.

### 3.4 Analysis of scheme selection results

The reservoir operation scheme decision-making model based on IWGAN-IWOA-CNN proposed in this paper is used to evaluate and select the schemes in the data set. Select the scheme selection results of some typical test data in data sets 1-3 for empirical analysis. **Table 6** shows the evaluation results of some schemes in dataset 1. **Figure 6** shows the evaluation results of some schemes in dataset 1-3. In order to intuitively compare the evaluation indexes of the scheme selection results, taking some reservoir operation schemes in dataset 1 as examples (schemes 1-24 in **Table 6**), the comparison diagram of scheme selection results is drawn as shown in **Figure 7**.

**Table 6:** Selection results of some schemes in dataset 1.

Scheme	Pre discharge flow (m3/s)	Pre discharge time (H)	Comprehensive evaluation value (true value)	IWGAN-IWOA-CNN predicted value	Relative error
1	130	12	0.429732	0.434281	1.0586%
2		24	0.451181	0.453907	0.6042%
3		32	0.484985	0.484338	-0.1334%
4		48	0.524055	0.521989	-0.3942%
5		60	0.565913	0.56401	-0.3363%
6		72	0.608977	0.604922	-0.6659%
7	200	12	0.468266	0.467083	-0.2526%
8		24	0.450929	0.455328	0.9755%
9		32	0.451549	0.454351	0.6205%
10		48	0.468626	0.469498	0.1861%
11		60	0.491939	0.491234	-0.1433%
12		72	0.51572	0.514425	-0.2511%
13	250	12	0.528245	0.528644	0.0755%
14		24	0.507876	0.510904	0.5962%
15		32	0.478134	0.482118	0.8332%
16		48	0.46102	0.46253	0.3275%
17		60	0.464031	0.465218	0.2558%
18		72	0.47142	0.472049	0.1334%
19	300	12	0.515353	0.51727	0.3720%
20		24	0.484222	0.487031	0.5801%
21		32	0.445025	0.449805	1.0741%
22		48	0.417365	0.424651	1.7457%
23		60	0.405425	0.41358	2.0115%
24		72	0.401801	0.412333	2.6212%

**Figure 6:**
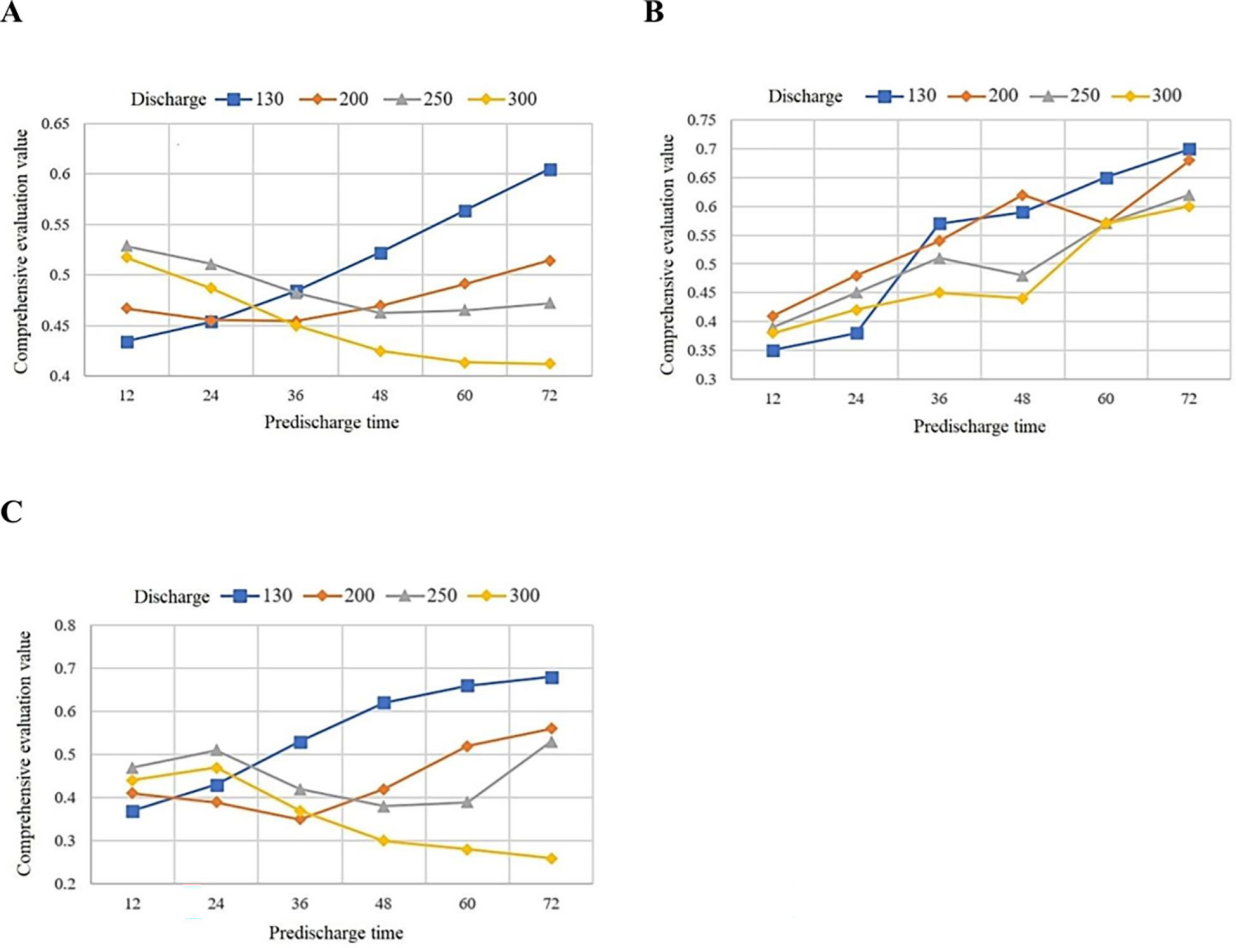
Comparison of different pre discharge schemes under different pre discharge time in dataset 1-3. **(A)** Dataset 1. **(B)** Dataset 2. **(C)** Dataset 3.

**Figure 7:**
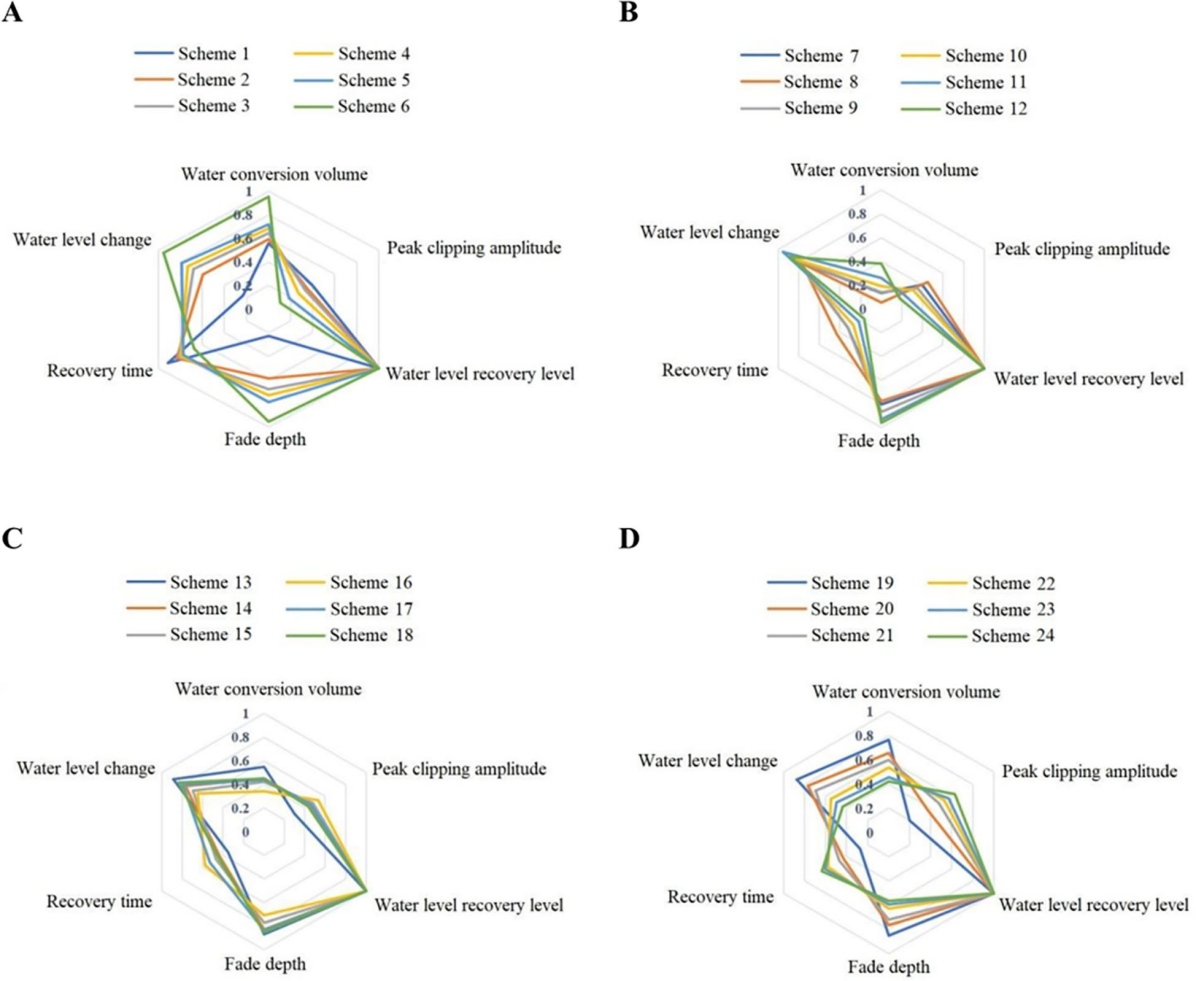
Radar chart of decision results of different schemes in dataset 1. **(A)** Scheme with pre discharge flow of 130. **(B)** Scheme with pre discharge flow of 200. **(C)** Scheme with pre discharge flow of 250. **(D)** Scheme with pre discharge flow of 300.

According to **Table 6**, compared with the actual comprehensive evaluation value of reservoir operation scheme, the relative error of IWGAN-IWOA-CNN model prediction is within ± 3%, indicating that the generalization ability and robustness of the model are good, and the result of scheme selection is ideal. According to the analysis of **Figure 6**:

(A) For the 10-year flood of Xidayang reservoir (dataset 1):

(1) When the reservoir operation scheme with pre discharge flow of 130 *m* ^3^/*s* is adopted, the comprehensive evaluation value of the scheme increases gradually with the increase of pre discharge time. When the reservoir operation scheme with pre discharge flow of 300 is adopted, the comprehensive evaluation value of the scheme gradually decreases with the increase of pre discharge time.

(2) When the pre discharge time is 36h, the evaluation results of each reservoir operation scheme have little difference, and the reservoir operation scheme with pre discharge flow of 130 is slightly better than that with pre discharge flow of 300 *m* ^3^/*s*, 250 *m* ^3^/*s* and 200 *m* ^3^/*s*.

(3) When the pre discharge time exceeds 48h, the scheme with pre discharge flow of 130 is significantly better than that with pre discharge flow of 200 *m* ^3^/*s*, 250*m* ^3^/*s* and 300 .

(B) For the 20-year flood of Xidayang reservoir (dataset 2):

(1) With the increase of pre discharge time, the comprehensive evaluation value curve of the schemes with pre discharge flow of 130 *m* ^3^/*s*., 200 *m* ^3^/*s*., 250 *m* ^3^/*s*. and 300 *m* ^3^/*s*. shows an increasing trend except that there is a downward trend at individual time points. The reservoir operation scheme with pre discharge time of 72 hours performs better.

(2) When the pre discharge time is between 24h and 36h, the scheme with pre discharge flow of 130 *m* ^3^/*s*. has the largest increase in the comprehensive evaluation value curve, indicating that the increase of pre discharge time has a certain effect on improving the effect of scheme selection.

(C) For the flood with a 50-year return period of Xidayang reservoir (dataset 3):

(1) With the increase of pre discharge time, the comprehensive evaluation curve of the scheme with pre discharge flow of 130 *m* ^3^/*s*. shows a continuous growth trend, the scheme with pre discharge flow of 250 *m* ^3^/*s*. shows a trend of first rising, then falling and then rising, and the scheme with pre discharge flow of 250 *m* ^3^/*s*. shows a trend of first falling and then rising. No matter how the trend of the three changes, the scheme with pre discharge time of 72 hours has the best effect.

(2) The comprehensive evaluation value of the scheme with pre discharge flow of 300m ^ 3/s increases first and then decreases with the increase of pre discharge time, and the reservoir operation scheme with pre discharge time of 24h performs best.

**Figures 7A–D** correspond to the four scheme selection curves of dataset 1 in **Figure 6**. From the perspective of evaluation indicators, except that the “Peak clipping amplitude” and “Recovery time” are cost indicators (the smaller the better), other evaluation indicators are benefit indicators (the larger the better). It can be seen from the radar chart that when the scheme with pre discharge flow of 130 *m* ^3^/*s*. and 200 *m* ^3^/*s*. is adopted, the peak clipping amplitude and recovery time of scheme 6 are relatively minimum, and the other evaluation indexes are relatively maximum, so it is more appropriate to choose the reservoir operation scheme with pre discharge time of 72 hours; When the pre discharge flow is 250 *m* ^3^/*s*. and 300 *m* ^3^/*s*., the reservoir operation scheme with pre discharge time of 12h is more appropriate.

## 4 Conclusion

Aiming at the complex decision-making problem of reservoir operation scheme, this paper proposes a reservoir operation scheme decision-making model based on IWGAN-IWOA-CNN. Firstly, the training samples of CNN are constructed by fuzzy optimization theory; Secondly, IWGAN is proposed to augment the data set of the original reservoir operation scheme. The algorithm uses the loss function which integrates Wasserstein distance, gradient penalty and difference item, and dynamically adds random noise in the process of model training. The experimental results show that the data generated by IWGAN has certain characterization ability; Then IWOA is proposed. The initial population of Logistic chaotic map, nonlinear convergence factor, adaptive weight and Levy flight disturbance strategy are introduced. The algorithm is compared with WOA, PSO, CS and GWO on 11 test functions. The experimental results show that IWOA has faster convergence speed, higher convergence accuracy and better stability; Finally, IWOA-CNN algorithm is proposed to optimize the CNN super parameters, and the optimal parameters are used to predict the model. The experimental results show that the prediction accuracy and scheme selection accuracy of the model in this paper are higher.

## Data availability statement

The raw data supporting the conclusions of this article will be made available by the authors, without undue reservation.

## Author contributions

QH, H-XH, and Z-ZL designed and performed the experiments. Z-HC, and YZ devised the experiments. QH, Z-HC helped with the data analysis and writing of the manuscript. All authors contributed to the article and approved the submitted version.
