# Supplementary material for: A decision-making method for reservoir operation schemes based on deep learning and whale optimization algorithm
Source: Front Plant Sci. 2023 Mar 24;14:1102855. doi: 10.3389/fpls.2023.1102855 (PMC10079899; doi:10.3389/fpls.2023.1102855)
Supplement: Supplementary file 1 [file DataSheet_1.docx]

Supplementary Material

# Supplementary Figures


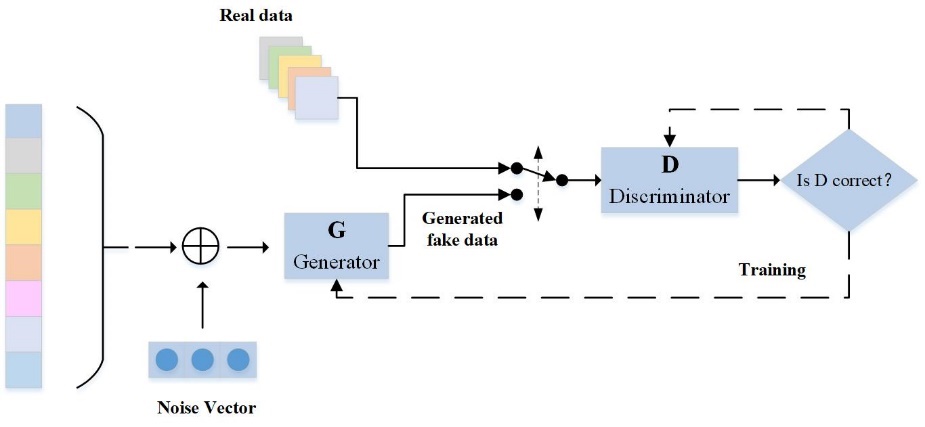


Figure 1. Basic structure of generative adversarial network.


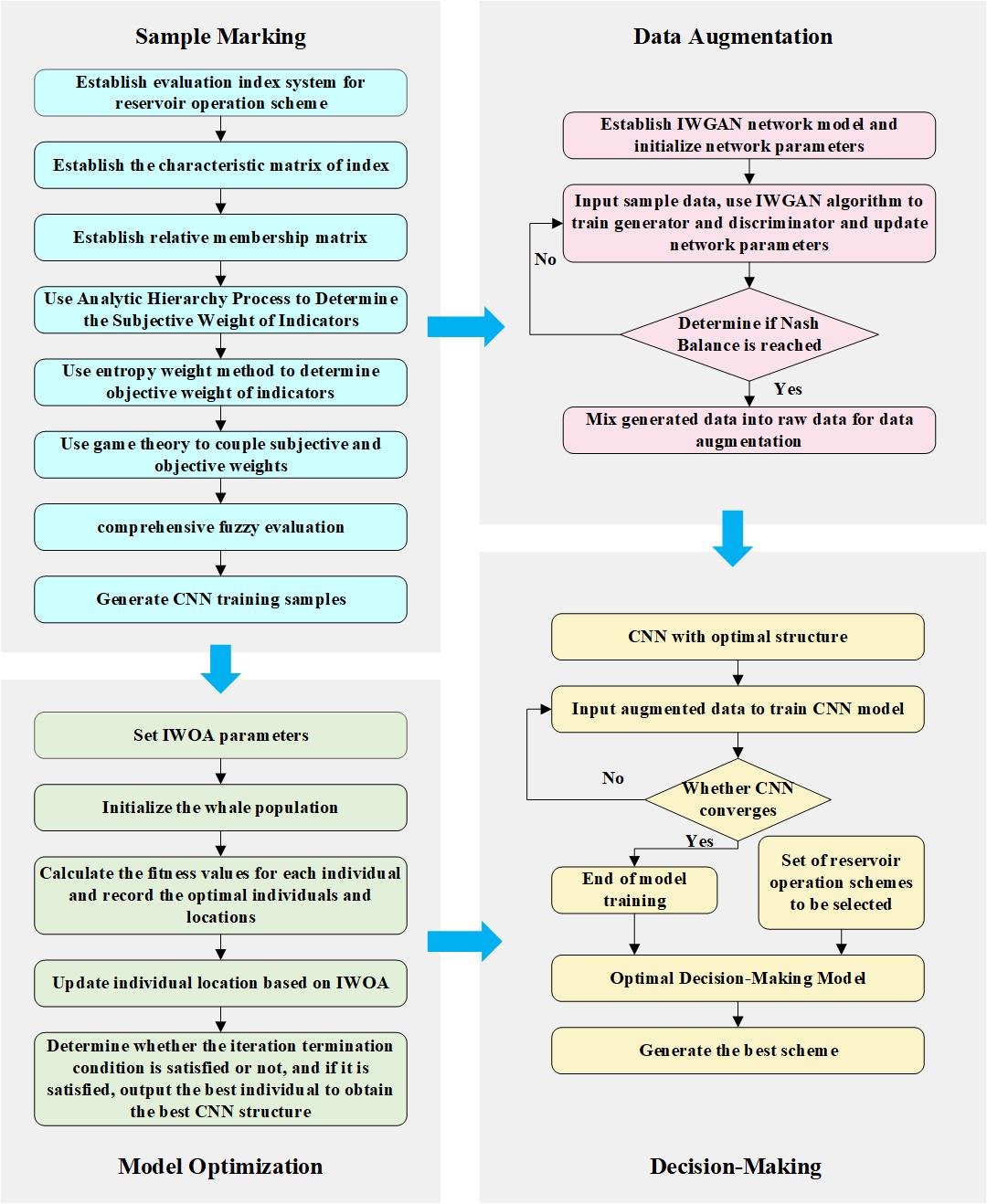


Figure 2. The framework of the decision-making method for reservoir operation schemes.


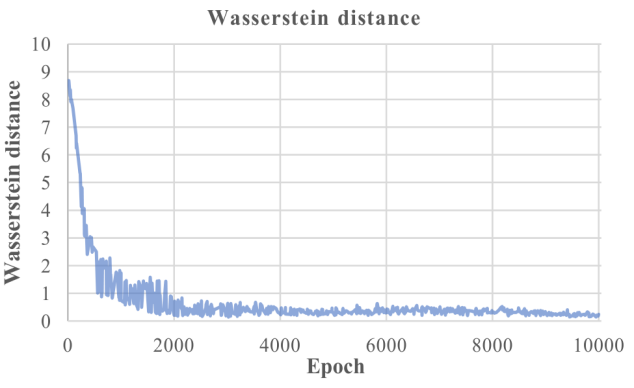


Figure 3. Wasserstein distance change in dataset 1.

| A 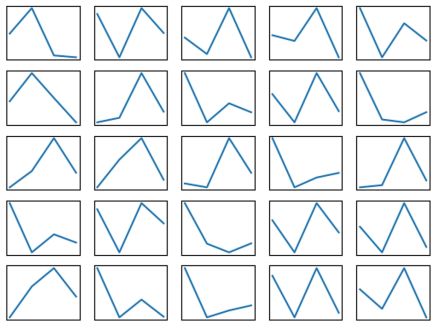 | B 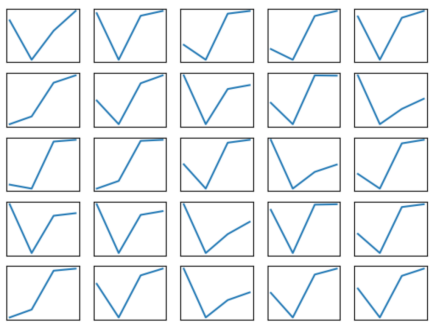 | C 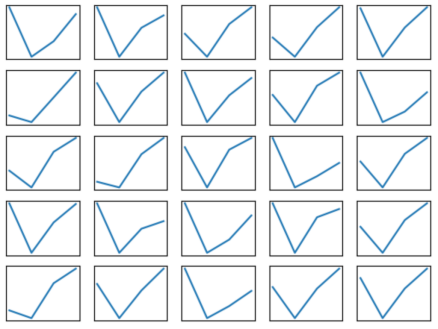 |
| --- | --- | --- |
|  | D 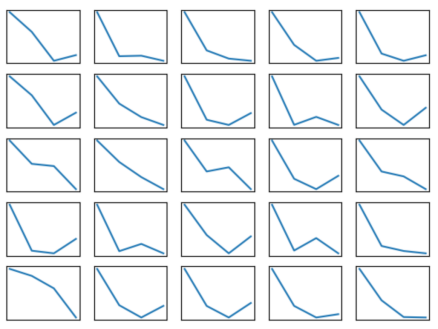 | E 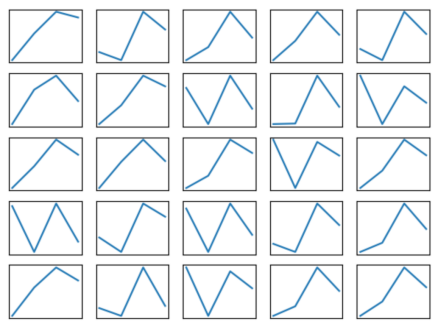 |

Figure 4. The real sample data and the generated sample data are randomly sampled, and the generated sample data are saved every 50 epochs. (A) Real sample data. (B) Generated sample data when e=50. (C) Generated sample data when e=1000. (D) Generated sample data when e=2850. (E) Generated sample data when e=4000.

| **A** 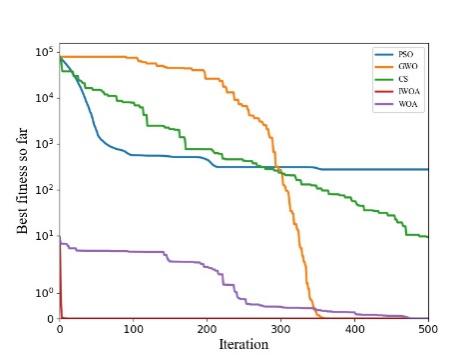 | **B** 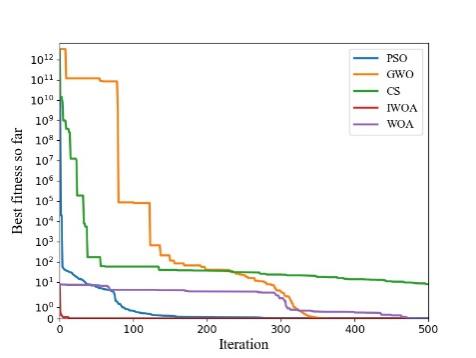 | **C** 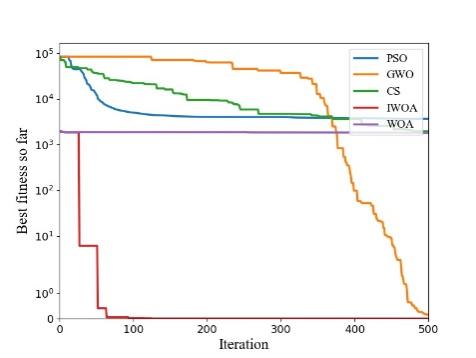 |
| --- | --- | --- |
| **D** 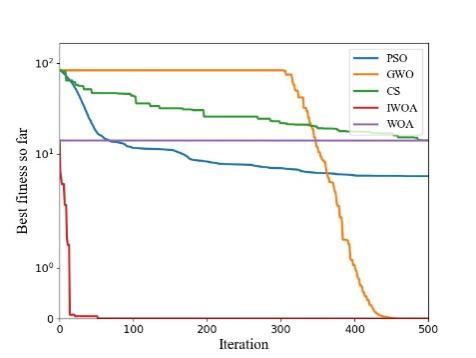 | **E** 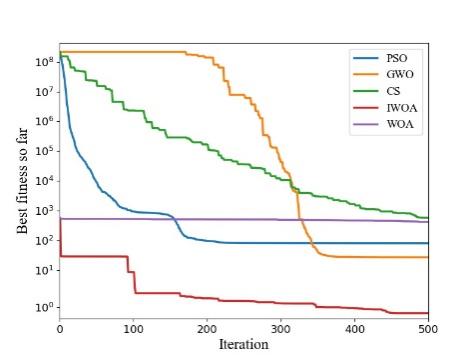 | **F** 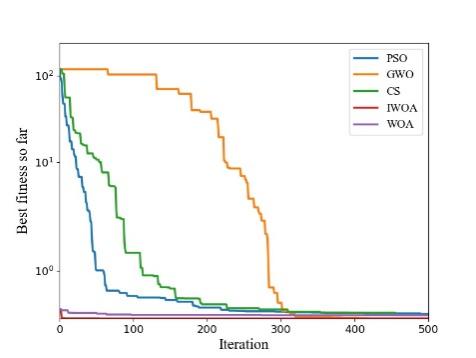 |
| **G** 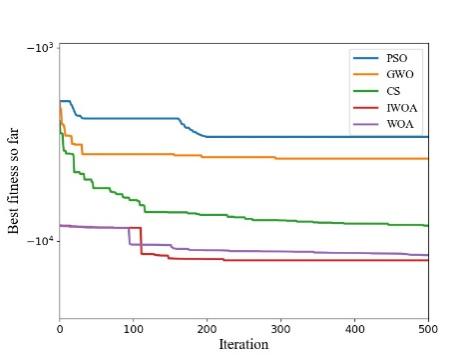 | **H** 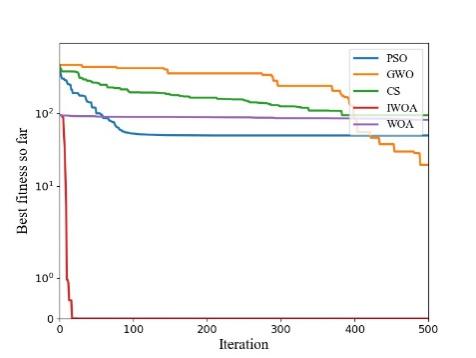 | **I** 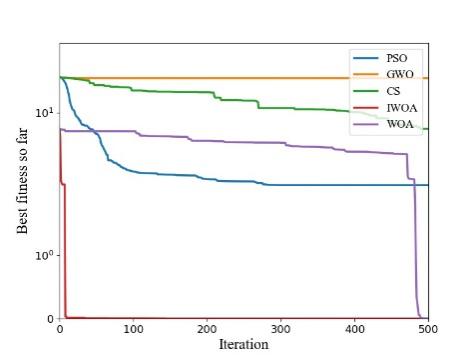 |
| **J** 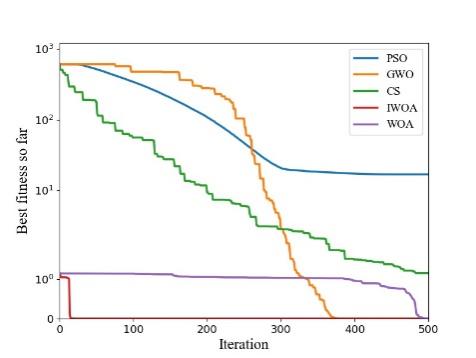 | **K** 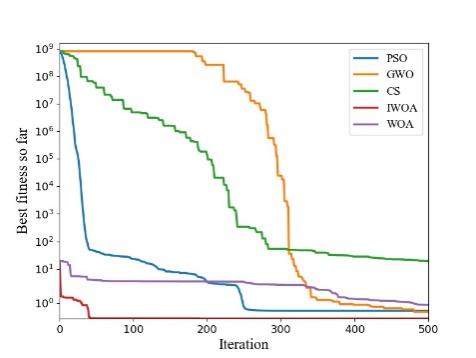 |  |

**Figure 5.** Convergence curves of different optimization algorithms on 11 test functions, A~K correspond to *f*_1_~*f*_11_ respectively.

| **A**  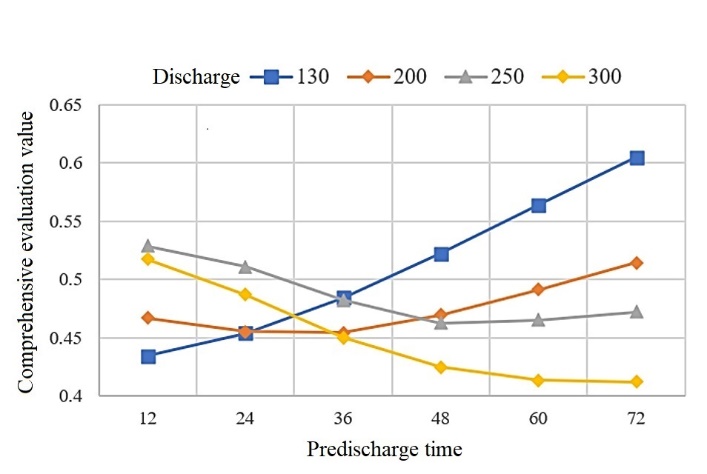 | **B**  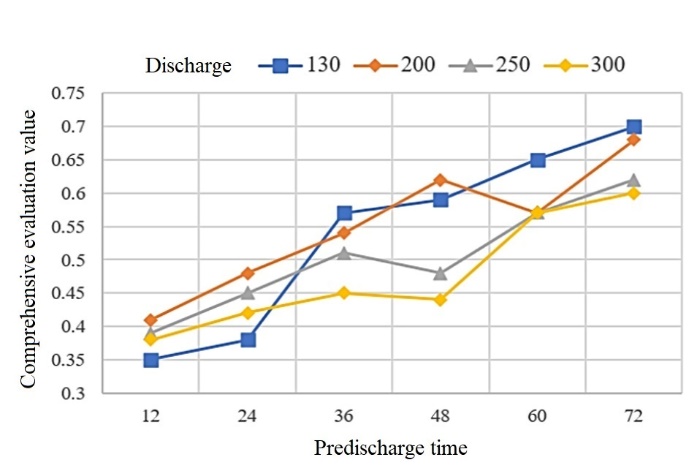 | |
| --- | --- | --- |
| **C**  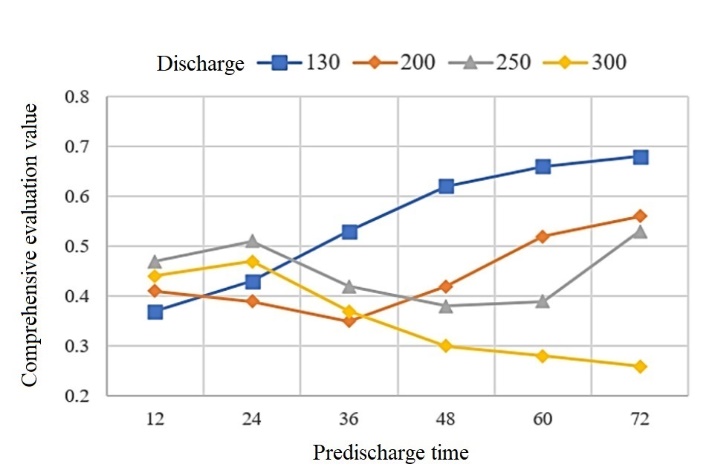 | |  |

**Figure 6.** Comparison of different pre discharge schemes under different pre discharge time in dataset 1-3. **(A)** Dataset 1. **(B)** Dataset 2. **(C)** Dataset 3.

| **A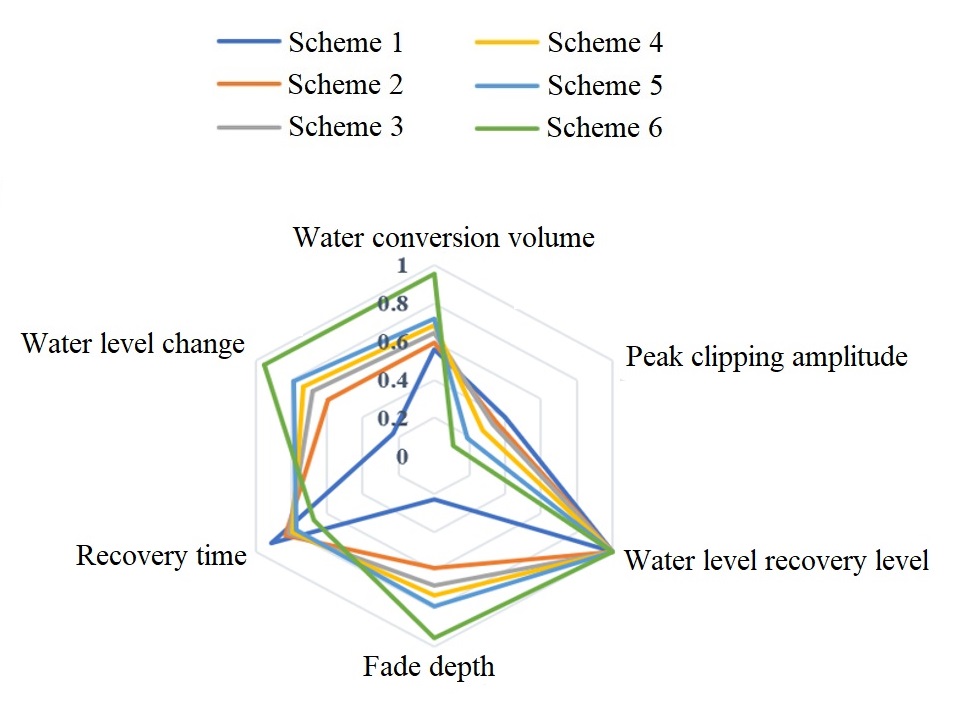** | **B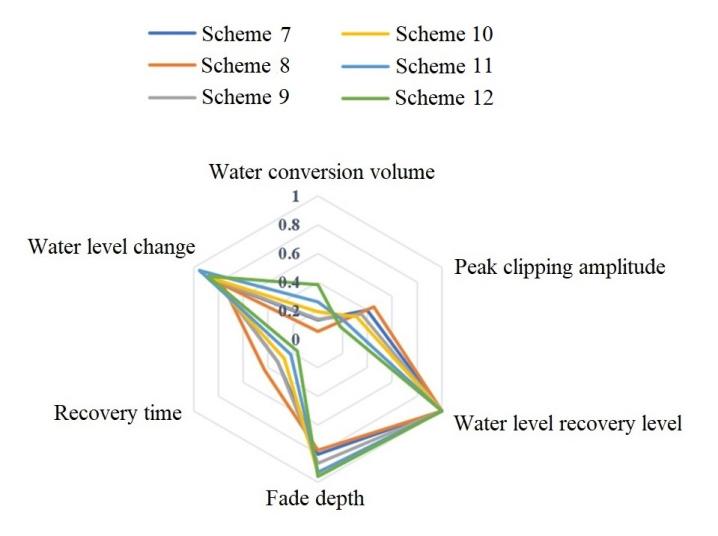** |
| --- | --- |
| **C**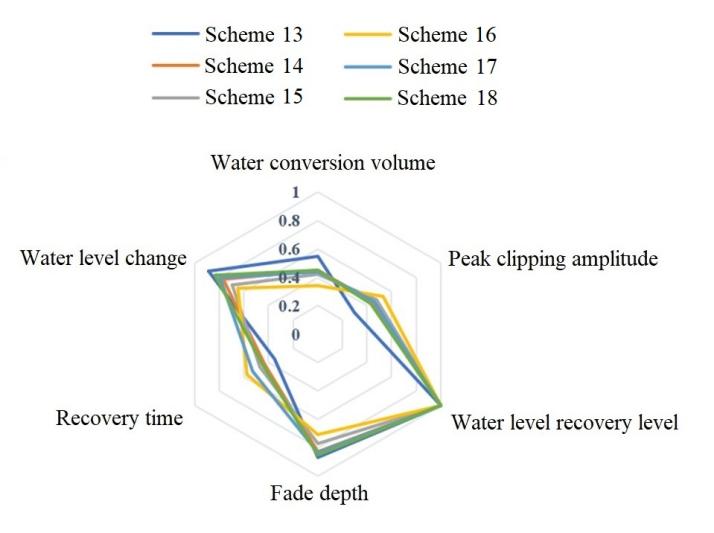 | **D**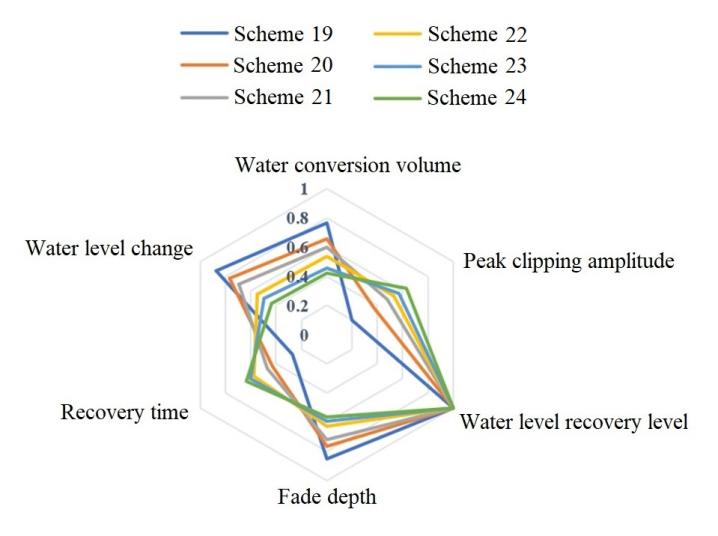 |

**Figure 7.** Radar chart of decision results of different schemes in dataset 1. **(A)** Scheme with pre discharge flow of 130$m^{3}/s$. **(B)** Scheme with pre discharge flow of 200$m^{3}/s$. **(C)** Scheme with pre discharge flow of 250$m^{3}/s$. **(D)** Scheme with pre discharge flow of 300$m^{3}/s$.
