# Supplementary material for: A decision-making method for reservoir operation schemes based on deep learning and whale optimization algorithm
Source: Front Plant Sci. 2023 Mar 24;14:1102855. doi: 10.3389/fpls.2023.1102855 (PMC10079899; doi:10.3389/fpls.2023.1102855)
Supplement: Supplementary file 2 [file DataSheet_2.docx]

**Appendix A. The flow chart of IWGAN.**

Appendix Figure 1 shows the flow chart of IWGAN.

Appendix Figure 1. The flow chart of IWGAN.

**Appendix B.** **The steps and the flow chart of IWOA-CNN.**

The steps of IWOA-CNN are as follows and Appendix Figure 2 shows the flow chart of IWOA-CNN:

Step 1: Set the relevant parameters of the IWOA-CNN algorithm, including the whale population size N, the number of iterations t, and the spatial dimension Dim (the dimension is determined by the optimization range of the parameters);

Step 2: Encode the CNN hyperparameters to be optimized into whale individuals in the IWOA-CNN algorithm in the form of real numbers, each individual as a structure of CNN, and then use the Logistic chaotic mapping method to initialize the population;

Step 3: Initialize CNN weights randomly, and select the root mean square error MSE as the fitness function value to calculate individual fitness;

Step 4: Update the individual according to the IWOA algorithm, and determine p_ best (i.e. the best CNN parameters) and g_ best (i.e. the best CNN structure);

Step 5: Judge whether the iteration termination condition is satisfied. If it is satisfied, output the optimal individual g_ Best, otherwise return to step 3 to continue.

Appendix Figure 2. The flow chart of IWOA-CNN.

**Appendix C. The network structure of generator and discriminator.**

The generator uses a 7-layer network structure, and the input of the network is a 6-dimensional implicit variable. Since the reservoir dispatch plan data is one-dimensional, the data dimension is expanded using the sampling layer 1D and reduced using the convolution layer 1D. The output convolution layer of the generator network uses the Tanh activation function, while the remaining network layers use the LeakyReLU function to prevent the gradient from disappearing. The size and number of convolution nuclei are determined by a large number of experiments. The generator uses four layers of convolution. The convolution cores are 3, 3, 3, 5, and the number of convolution cores is 64, 32, 16, 6. The number of output convolution cores is consistent with the evaluation index data dimension of the reservoir dispatch plan. The network structure of the generator is shown in Appendix table 1.

Appendix table 1 The network structure of IWGAN generator

| Layer | Layer name | Index | Parameters |
| --- | --- | --- | --- |
| 1 | Full connection layer | Number of neurons | 128×6 |
|  |  | Activation function | LeakyReLU |
| 2 | Up Sampling Layer 1D | Up Sampling Factor | 2 |
| 3 | Convolution Layer 1D | Convolution Kernel Size | 3 |
|  |  | Number of convolution cores | 64 |
|  |  | Step | 1 |
|  |  | Activation function | LeakyReLU |
| 4 | Up Sampling Layer 1D | Sampling Factor | 2 |
| 5 | Convolution Layer 1D | Convolution Kernel Size | 3 |
|  |  | Number of convolution cores | 32 |
|  |  | Step | 1 |
|  |  | Activation function | LeakyReLU |
| 6 | Convolution Layer 1D | Convolution Kernel Size | 3 |
|  |  | Number of convolution cores | 16 |
|  |  | Step | 1 |
|  |  | Activation function | LeakyReLU |
| 7 | Convolution Layer 1D | Convolution Kernel Size | 5 |
|  |  | Number of convolution cores | 6 |
|  |  | Step | 1 |
|  |  | Activation function | Tanh |

The discriminator uses an eight-layer network structure, which consists of three convolution layers and two maximum pooling layers. The convolution kernel size is always 3, the number of convolution cores is 32, 64 and 128, the pooling layer decreases by 2, the activation function is used the same as the generator, and Flatten layer is added after the last pooling operation, and Full connection layer and Dropout layer are added after the last convolution operation to prevent fitting. The output of the discriminator network represents the probability that the input data is real data, so the activation function does not use the Sigmoid function but uses the Tanh activation function. The network structure of the discriminator is shown in Appendix table 2.

Appendix table 2 The network structure of IWGAN discriminator

| Layer | Layer name | Index | Parameters |
| --- | --- | --- | --- |
| 1 | Convolution Layer 1D | Convolution Kernel Size | 3 |
|  |  | Number of convolution cores | 32 |
|  |  | Step | 1 |
|  |  | Activation function | LeakyReLU |
| 2 | Maximum Pooling Layer | Sampling Window | 2 |
| 3 | Convolution Layer 1D | Convolution Kernel Size | 64 |
|  |  | Number of convolution cores | 1 |
|  |  | Step | LeakyReLU |
|  |  | Activation function | 3 |
| 4 | Maximum Pooling Layer | Sampling Window | 2 |
| 5 | Convolution Layer 1D | Convolution Kernel Size | 3 |
|  |  | Number of convolution cores | 128 |
|  |  | Step | 1 |
|  |  | Activation function | LeakyReLU |
| 6 | Full connection layer | Number of neurons | 64 |
|  |  | Activation function | LeakyReLU |
| 7 | Dropout Layer | Dropout Probability | 0.4 |
| 8 | Full connection layer | Number of neurons | 1 |
|  |  | Activation function | Tanh |

**Appendix D. The training process of IWGAN.**

The training process of IWGAN model is divided into pre-training and formal training. The purpose of pre-training is to ensure that the loss function of the discriminator can guide the training of the generator more accurately during formal training.

(1) Pre-training: first, the root mean square error is used as the loss function of the generator to train. As the number of training increases, the loss function tends to decrease, and then the sample data generated by the generator continuously approximates the true data distribution. The more similar the generated sample data is to the real sample data, the more accurate the subsequent discriminator training will be.

(2) Formal training: after the pre-training is completed, the generator and discriminator are formally alternated for antagonistic training. First, a random sample is taken from the Gauss noise, and a set of sampled noise is input into the generator to get a set of generated sample data. At this time, the generator has not started training. Then, a set of real sample data is randomly sampled and input into the discriminator with the sample data just generated for training. Adam optimization algorithm is used to update the network parameters of the discriminator and minimize the loss function during training. The generator is then trained, the discriminator is fixed, and the network parameters are updated by calculating the generator's loss function. The discriminator is trained five times and the generator is trained one time.

At the beginning of the training, the distribution of the sample data generated by the generator is very similar to that of the real sample data, and the discriminator can easily distinguish between true and false. As training progresses, the sample data generated by the generator approximates the true data distribution more and more, making it difficult for the discriminator to judge. Generators and discriminators are trained against the game to reach the Nash balance. The distance between the real data and Wasserstein that generates the distribution of the data is close to 0, and the training of the whole IWGAN model ends here.

**Appendix E. Optimal CNN Model Structure**

The relationship between the structure of CNN model (such as the number of convolution layers, pooled layers, convolution core size, number of convolution cores, etc.) and its performance is explored through a large number of experiments. The optimal CNN model structure is determined, and the relevant parameters are shown in Appendix table 3.

Appendix table 3 Optimal CNN Model Structure

| Layer (type) | Output Shape | Parameters |
| --- | --- | --- |
| conv1d_1(Conv1D) | (None,6,8) | 32 |
| max_pooling1d_1(MaxPooling1D) | (None,3,8) | 0 |
| conv1d_2(Conv1D) | (None,3,16) | 400 |
| max_pooling1d_2(MaxPooling1D) | (None,1,16) | 0 |
| flatten_1(Flatten) | (None,16) | 0 |
| dense_1(Dense) | (None,15) | 255 |
| dropout_1(Dropout) | (None,15) | 0 |
| activation_1(Activation) | (None,15) | 0 |
| dense_2(Dense) | (None,1) | 16 |
